# The Antioxidant and Xanthine Oxidase Inhibitory Activity of *Plumeria rubra* Flowers

**DOI:** 10.3390/molecules23020400

**Published:** 2018-02-13

**Authors:** Siti Sarwani Putri Mohamed Isa, Abdulwali Ablat, Jamaludin Mohamad

**Affiliations:** Institute of Biological Sciences, Faculty of Science, University of Malaya, Kuala Lumpur 50603, Malaysia; sarwaniputri@gmail.com (S.S.P.M.I.); aablat@gmail.com (A.A.)

**Keywords:** xanthine oxidase, *Plumeria rubra*, antioxidant, phenol, flavonoid

## Abstract

*Plumeria rubra* Linn of the family Apocynaceae is locally known in Malaysia as “Kemboja”. It has been used by local traditional medicine practitioners for the treatment of arthritis-related disease. The LCMS/MS analysis of the methanol extract of flowers (PR-ME) showed that it contains 3-*O*-caffeyolquinic acid, 5-caffeoquinic acid, 1,3-dicaffeoquinic acid, chlorogenic acid, citric acid, 3,3-di-*O*-methylellagic acid, kaempferol-3-*O*-glucoside, kaempferol-3-rutinoside, kaempferol, quercetin 3-*O*-α-l-arabinopyranoside, quercetin, quinic acid and rutin. The flower PR-ME contained high amounts of phenol and flavonoid at 184.632 mg GAE/g and 203.2.2 mg QE/g, respectively. It also exhibited the highest DPPH, FRAP, metal chelating, hydrogen peroxide, nitric oxide superoxide radical scavenging activity. Similarly, the XO inhibitory activity in vitro assay possesses the highest inhibition effects at an IC_50_ = 23.91 μg/mL. There was no mortality or signs of toxicity in rats at a dose of 4 g/kg body weight. The administration of the flower PR-ME at doses of 400 mg/kg to the rats significantly reduced serum uric acid 43.77%. Similarly, the XO activity in the liver was significantly inhibited by flower PR-ME at doses of 400 mg/kg. These results confirm that the flower PR-ME of *P. rubra* contains active phytochemical compounds as detected in LCMS/MS that contribute to the inhibition of XO activity in vitro and in vivo in reducing acid uric level in serum and simultaneously scavenging the free radical to reduce the oxidative stress.

## 1. Introduction

Gout is a hyperuricemia, due to the elevated uric acid above the normal level leading to the formation of monosodium urate (MSU) crystals within joints and subcutaneous tissues which is associated to the development of painful attacks of gouty arthritis [1]. The formation of uric acid occurs from the final biosynthesis in the purine catabolic pathway which it responsible for catalyzing the oxidation process of hypoxanthine to xanthine and further, xanthine to uric acid. The overproduction and/or under excretion of uric acid lead to the major contributor of incidence of gout [2]. The elevation of oxidative stress has been reported in gouty patients [3]. Xanthine oxidase (XO) is sn important biological source of oxygen-derived free radicals which contribute to oxidative damage to living tissues involved in many pathological processes including inflammation, atherosclerosis, cancer, aging and gout [4,5,6]. Some bioactive compounds possess the ability to inhibit the activity of XO that leads to a decrease in uric acid levels and reactive oxygen species (ROS) production. This will help to reduce inflammation, hyperuricemia and antioxidative effects. Allopurinol is a known XO inhibitor commonly used to lower urate levels in serum and urine. It is also effective in decreasing flares and tophi when urate levels are reduced. Although allopurinol has been successfully used to reduce uric acid level but it is still not the right drug for the treatment of acute gouty arthritis [7]. Allopurinol generates superoxide [8] and can develop undesirable symptoms in persons that are allergic to allopurinol [9]. Several severe reactions can occur, including liver function abnormalities [10], a fatal complication known as “allopurinol hypersensitivity syndrome” [9,11] and adverse drug reactions such as Toxic Epidermal Necrolysis syndrome (TENS) [6]. Allopurinol also contributes to adverse effects including gastrointestinal toxicity, renal toxicity or gastrointestinal bleeding [12]. Numerous studies aiming to develop an alternative medicine for the treatment of hyperuricemia and gout have focused on plant natural phytochemicals without adverse side effects. Xanthine oxidase inhibitors derived from plant natural sources have been reported [13,14]. Flavonoids have been reported to possess the ability to act as active inhibitors of XO [5]. They also act as free radical scavengers by donating hydrogen atoms to free radicals. *Plumeria rubra* contains high amount of flavonoids and could be used as a new alternative to allopurinol with increased therapeutic activity and fewer side effects. In the present study, *P. rubra* flowers were selected based on their ethnomedicinal use in gout and arthritis [15], inflammation [16], constipation [17] and hypolipidemia [18]. Despite the widely popular use of *P. rubra* flowers, available scientific information on the potential effects of *P. rubra* flowers in animal models of gout is limited. There is no previous research reporting XO inhibitory activity of *P. rubra* making it is worthwhile to evaluate the inhibitory effects of bioactive compounds derived from the red flowers of *P. rubra.* The main objectives the present study were thus to evaluate the activity of flower extract of *P. rubra* as a XO inhibitor in an in vitro assay and in in vivo animal models for gout treatment. 

## 2. Results

### 2.1. Liquid Chromatography Mass Spectrometry Combined with Mass Spectrometry (LCMS/MS)

**Figure 1:**
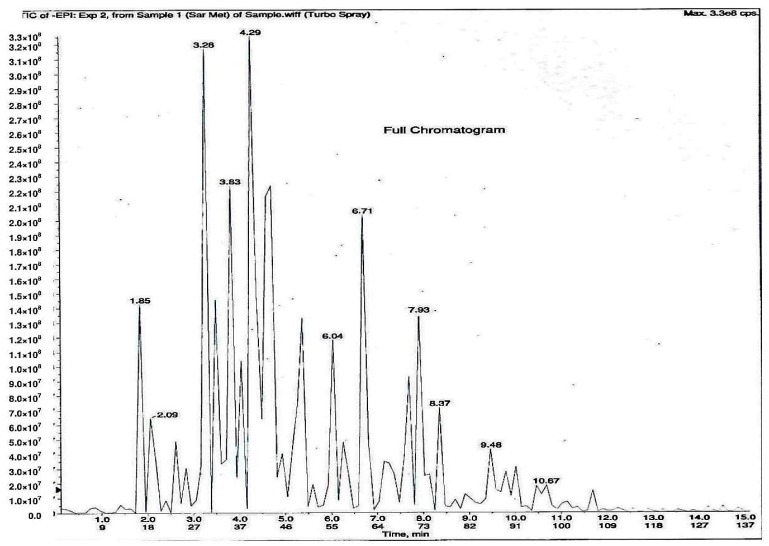
LCMS/MS chromatogram of PR-ME extract from red flower of *Plumeria rubra*. **1**: 3-*O*-Caffeoylquinic acid; **2**: 5-*O*-Caffeoylquinic acid; **3**: Chlorogenic acid; **4**: Citric acid; **5**: Kaempferol-3-*O*-glucoside; **6**: Kaempferol-3-rutinoside; **7**: Kaempferol; **8**: Quercetin 3-*O*-α-l-arabinopyranside; **9**: Quercetin; **10**: Quinic acid; **11**: Rutin.

As shown in Figure 1, 11 phytochemical compounds were detected in the methanol flower extract of *P. rubra* by LCMS/MS, including 3-*O*-caffeoylquinic acid, 5-*O*-caffeoylquinic acid, chlorogenic acid, citric acid, kaempferol-3-*O*-glucoside, kaempferol-3-rutinoside, kaempferol, quercetin 3-*O*-α-l-arabinopyranside, quercetin, quinic acid and rutin. This suggested that these compounds contributed to the antioxidant and xanthine oxidase inhibitory activities in the *Plumeria rubra* flower extracts.

### 2.2. Determination of Total Phenol and Flavonoid Contents from P. rubra Flower Extracts

The methanol extract (PR-ME) showed the highest TFC and TFC followed by water extract (PR-WE), chloroform extract (PR-CE) and hexane extract (PR-HE) (Table 1). 

### 2.3. DPPH Radical Scavenging Activity Assay

As shown in Table 2, the IC_50_ value of PR-ME extract was the highest in DPPH, FRAP, metal chelating, nitric oxide and superoxide scavenging activities. This gives an indication that PR-ME extract possesses a strong antioxidant potential to scavenge free radicals.

### 2.4. Xanthine Oxidase Inhibitory Activity In Vitro Assay

**Figure 2:**
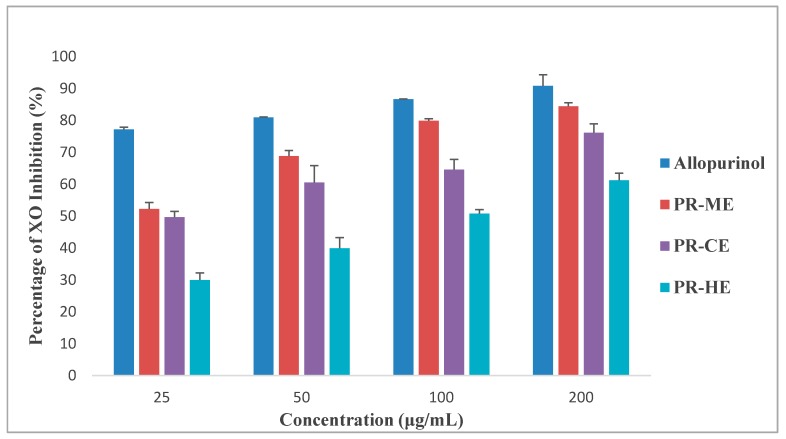
Percentage xanthine oxidase (XO) inhibitory activity of *P. rubra* flower extract in vitro. PR-ME; methanol extract; PR-WE; water extract; PR-CE: Chloroform extract; PR-HE: hexanre extract. Each value is represented as mean ± S.E. (*n* = 3).

Figure 2 illustrates the different effect of four different concentrations of PR-HE, PR-CE, PR-ME and PR-WE extract of *P. rubra* flower as compared to the standard positive control, allopurinol, in inhibition of xanthine oxidase enzyme. All four extracts showed XO inhibitory activity in a dose dependent manner and were capable of inhibiting XO-induced superoxide formation at the highest concentration tested, 200 μg/mL. The order of XO inhibition activity was allopurinol > PR-ME extract > PR-WE > PR-CE extract > PR-HE extract. The percentage of inhibition of XO activity by PR-HE, PR-CE, PR-ME and PR-WE extracts of *P. rubra* flowers increased with increasing concentration of the extracts. The PR-ME extract showed the strongest XO inhibitor with highest percentage of XO inhibition of 84.39% at 200 μg/mL.

Since the PR-ME extract disolayed significantly highest in vitro XO inhibitory activity than the other extracts, the PR-ME extract of *P. rubra* flower was selected for testing in an anti-hyperuricemic rat model in vivo and for xanthine oxidase inhibitory activity in serum and liver.

### 2.5. Acute Toxicity of Plumeria rubra Extract

An acute toxicity study showed that there was no lethality or any toxic behaviour in the rats. The PR-ME extract of *P. rubra* red flower did not show any mortality and none of rats of either sex showed any visible symptoms of toxicity up to a dose of 4 g/kg body weight, indicating a high margin of safety.

### 2.6. Effect PR-ME Extract in Hyperuricemic Induced Rat

**Figure 3:**
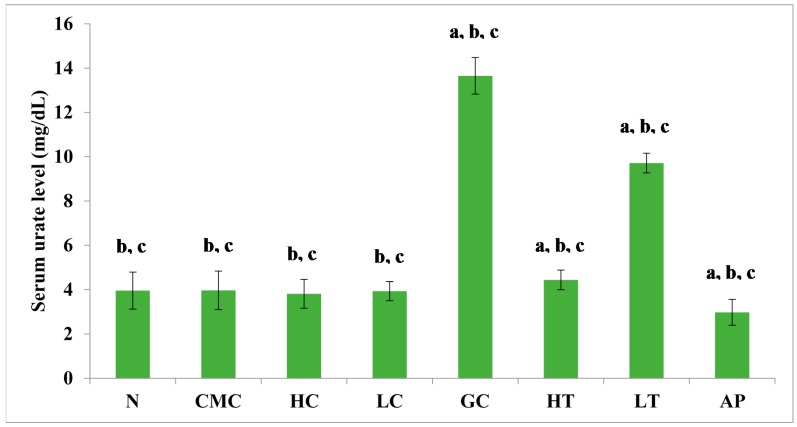
Effect of the PR-ME extract of *P. rubra* red flower on serum urate level in PO-induced hyperuricaemia in rats and control rats at 7 days. N: Rats without PO treatment nor treated with *P. rubra* extract, CMC: Vehicle group of rats, HC: Rats administered with high dose (400 mg/kg) *P. rubra* extract, LC: Rats administered with low dose (200 mg/kg) *P. rubra* extract, GC: Gout control induced with PO, HT: Hyperuricemic rats dosed with high dose (400 mg/kg) *P. rubra* extract, LT: Hyperuricemic rats dosed with low dose (200 mg/kg) *P. rubra* extract, AP: Hyperuricemic rats dosed with 10 mg/kg allopurinol. The data are representative of six animals and expressed as mean ± S.E. with a *p* < 0.05 significant when compared to normal (N) group, b *p* < 0.05 significance when compared to the hyperuricemic gout control (GC) group, c *p* < 0.05 significance when compared to the allopurinol (AP) group.

The PR-ME extract from *P. rubra* flower showed dose-dependent anti-hyperuricemic effects on serum urate levels in normal and hyperuricemic mice (Figure 3). The serum uric acid levels of the hyperuricemic gout-control group were significantly (*p* < 0.05) elevated to 13.65 ± 0.83 mg/dL as compared to the other group. No significant difference was observed in serum urate levels between the groups of normal rats and non-induced rats. In the hyperuricemic rats, after administration with PR-ME extract of *P. rubra* flower at doses of 200 and 400 mg/kg, serum and urate levels were significantly decreased in a dose-dependent manner as compared to the gout control group. The serum uric acid levels of hyperuricemic rats treated with high dose of 400 mg/kg PR-ME extract of *P. rubra* flower was significantly reduced to 4.44 ± 0.44 mg/dL. It can be seen that the dose dependent effects of PR-ME extract of *P. rubra* flower in reducing serum urate levels was seen in hyperuricemic induced rats rather than in the normal, CMC vehicle, high dose control, low dose group. The standard drug allopurinol markedly reduced the level of serum urate of hyperuricemic rats to values even lower than that seen in gout control rats with a concentration of serum urate level at 2.98 ± 0.58 mg/dL.

### 2.7. Effect of PR-ME Extract on XO Activity in Hyperuricemic Induced Rat

**Figure 4:**
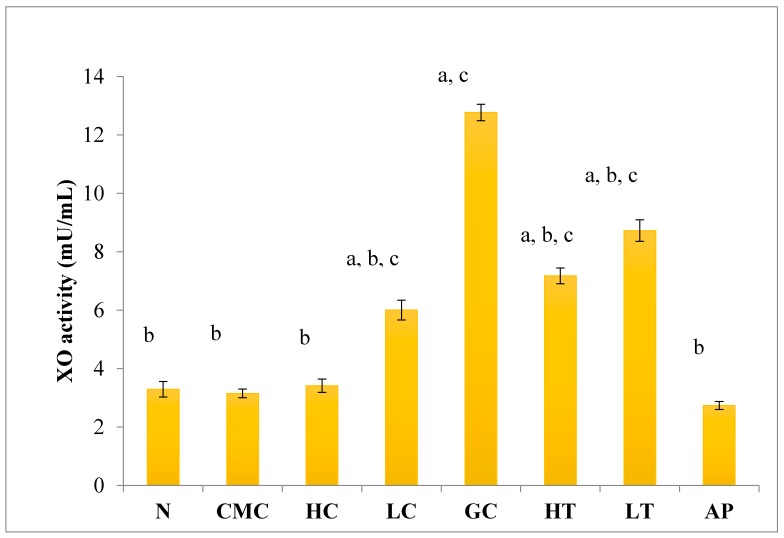
Effect of the PR-ME extract of *P. rubra* red flower on XO activity in serum of PO-induced hyperuricaemia in rat and normal control rat at 7 days. N: Rats not induced with PO or treated with *P. rubra* extract and standard drug allopurinol, CMC: Vehicle group of rats, HC: Rats administered with high dose (400 mg/kg) *P. rubra* extract, LC: Rats administered with low dose (200 mg/kg) *P. rubra* extract, GC: Hyperuricemic gout control induced with PO (280 mg/kg), HT: Hyperuricemic rat dosed with high dose (400 mg/kg) *P. rubra* extract, LT: Hyperuricemic rat dosed with low dose (200 mg/kg) *P. rubra* extract, AP: Hyperuricemic rat dosed with 10 mg/kg allopurinol. The data are representative of six animals and expressed as mean ± S.E. with a *p* < 0.05 significance when compared to the normal (N) group, b *p* < 0.05 significance when compared to the hyperuricemic gout control (GC) group c *p* < 0.05 significance when compared to the allopurinol (AP) group.

As shown in Figure 4, there was no significant difference in serum XO activity between the group of normal untreated extract and non-induced group. The administration of 400 mg/kg of PR-ME extract of *P. rubra* flower to the non-induced rats caused weak inhibition of XO in the serum. However, at low dose of 200 mg/kg of PR-ME extract of *P. rubra* flower, the XO is not inhibited and the XO activity is more than the normal value. It was noted that PO successfully play a role as a competitive uricase inhibitor since the XO activity in the hyperuricemic gout control group (GC) was significantly increased (*p* < 0.05) at a level of 12.77 ± 0.33 mU/mL. In hyperuricemic rats, after administration with PR-ME extract of red flowers at doses of 200 mg/kg and 400 mg/kg, the XO activity were significantly decreased in a dose-dependent manner as compared to the gout control group. The XO activity of hyperuricemic rats treated with high dose of 400 mg/kg PR-ME extract from *P. rubra* flower was reduced significantly to 7.18 ± 0.30 mU/mL 43.77%, whereas with dose of 200 mg/kg reduced the XO activity significantly at 8.73 ± 0.52 mU/mL( by 31.64%) as compared to the gout control group. There was no significant difference between the group of hyperuricemic PO-induced rats treated with high dose of 400 mg/kg PR-ME extract with the group of normal rats treated with 200 mg/kg PR-ME extract of *P. rubra* flower.

### 2.8. XO Inhibitory Activity in Liver

**Figure 5:**
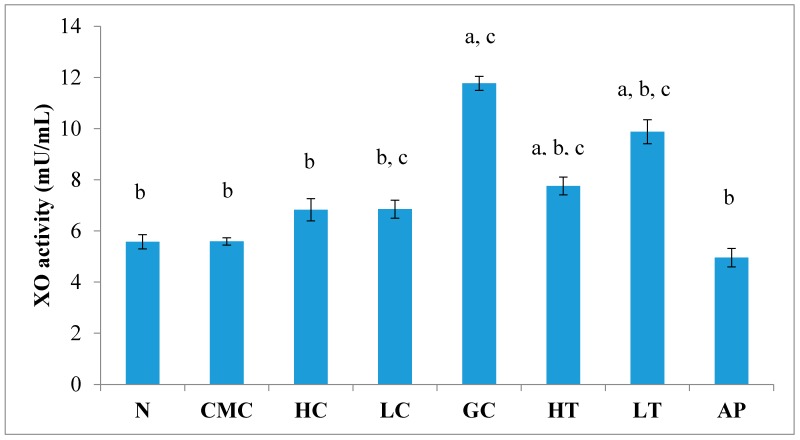
Effect of the PR-ME extracts of *P. rubra* flower on XO activity in liver PO-induced hyperuricaemia in rat and normal control rat at 7 days. N: Rats not treated with PO or with *P. rubra* extract, CMC: Vehicle group of rats, HC: Rats administered with high dose (400 mg/kg) *P. rubra* extract, LC: Rats administered with low dose (200 mg/kg) *P. rubra* extract, GC: Hyperuricemic gout control induced with PO, HT: Hyperuricemic rats dosed with high dose (400 mg/kg) *P. rubra* extract, LT: Hyperuricemic rat dosed with low dose (200 mg/kg) *P. rubra* extract, AP: Hyperuricemic rat dosed with 10 mg/kg allopurinol. The data are representative of six animals and expressed as mean ± S.E with a *p* < 0.05 significance when compared to the normal (N) group, b *p* < 0.05 significance when compared to the hyperuricemic gout control (GC) group, c *p* < 0.05 significance when compared to the allopurinol (AP) group.

The levels of uric acid depend on a XO-catalyzed reaction. As shown in Figure 5, there was no significant difference in liver XO activity between the normal untreated group and the non-induced group. The administration of PR-ME extract of *P. rubra* flower does not show any effect to XO activity of normal rats as these doses did not produced any inhibition of XO activity in the liver. Potassium oxonate (PO) is a uricase inhibitor that causes the gout control group to exhibit significantly increased XO activity in liver. However, the liver XO activity of the hyperuricemic rats was significantly decreased in a dose-dependent manner with the administration of *P. rubra* PR-ME extract. The standard drug allopurinol reduced significantly the XO inhibition by 57.86%.

### 2.9. The Effect of PR-ME Extract on Body Weight

**Figure 6:**
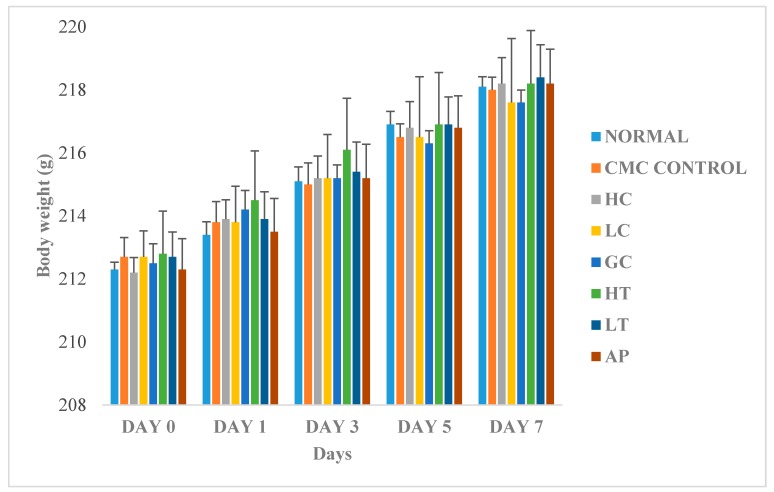
Effect of PR-ME extract of *P. rubra* red flower on body weight of rats. N: Rats not treated with PO, *P. rubra* extract, CMC Control: Vehicle group of rats, 0.5% CMC, HC: Rats administered with high dose (400 mg/kg) *P. rubra* extract, LC: Rats administered with low dose (200 mg/kg) *P. rubra* extract, GC: Gout control induced with PO, HT: Hyperuricemic rats dosed with high dose (400 mg/kg) *P. rubra* extract, LT: Hyperuricemic rats dosed with low dose (200 mg/kg) *P. rubra* extract, AP: Hyperuricemic rats dosed with 10 mg/kg allopurinol. The data are representative of six rats and expressed as mean ± S.E.

Figure 6 shows the changes in body weight between the normal and treated groups. The body weights of all the rats gradually increased throughout the experiment, regardless of their treatment group. Since there were no significant differences of body weight between the treatments groups, therefore, we conclude that the body weight of the rats was not influenced by extract of *P. rubra* flower. This indicated that the *P. rubra* flower extract is not toxic to the rats.

## 3. Discussion

The LCMS/MS analysis of PR-ME extract of *P. rubra* flowers showed the presence of 3-*O*-caffeyolquinic acid, 5-caffeoquinic acid, chlorogenic acid, citric acid, kaempferol-3-*O*-glucoside, kaempferol-3-rutinoside, kaempferol, quercetin 3-*O*-α-l-arabinopyranoside, quercetin, quinic acid and rutin. The high total phenol content in PR-ME extract is contributed to the presence of 3-caffeyolquinic acid, 5-caffeoquinic acid, chlorogenic acid and quinic acid. Similarly, the high total flavonoid contents in the PR-ME is contributed by the presence of kaempferol-3-*O*-glucoside, kaempferol-3-rutinoside, kaempferol, quercetin 3-*O*-α-l-arabinopyranoside, quercetin and rutin. This could be due to the ability of methanol to inhibit the reaction of polyphenol oxidase that causes the oxidation of phenolic compounds and its ease of evaporation as compared with water. The total phenolic contents and total flavonoid contents of PR-HE extract were much lower, in agreement with Sahreen et al. [19] and Medini et al. [20] as both of these groups indicated a low content of phenols and flavonoids in the hexane extract. Furthermore, the results also suggested that the extractability of polyphenols is influenced by the polarity and viscosity of the extraction solvent used [21,22].

The highest inhibition of DPPH radical in PR-ME is contributed to the presence of quercetin, kaempferol, rutin, 3-*O*-caffeyolquinic acid, 5-*O*-caffeoquinic acid, chlorogenic acid and quinic acid that were detected by LCMS/MS. The number and configuration of H-donating hydroxyl groups are the main structural features that influence the antioxidant capacity [23]. According to Wang et al. [24], the glycosylation of flavonol, kaempferol-3-glycoside, kaempferol-3-rutinoside and quercetin 3-*O*-α-l-arabinopyranoside on both OCH_3_ and OH groups reduces the DPPH free radical scavenging potential. Yang et al. [24] and Lue et al. [25] have reported that rutin has the capability to scavenge DPPH radicals. In the FRAP activity, the PR-ME was able to reduce ferrous ion that gives an indication it has the ability to scavenge the free radicals, contributed by the presence 3-*O*-caffeyolquinic acid, 5-*O*-caffeoquinic acid, 1,3-dicaffeoquinic acid, chlorogenic acid, 3,3-di-*O*-methyl-ellagic acid, kaempferol-3-*O*-glucoside, kaempferol-3-rutinoside, kaempferol, quercetin 3-*O*-arabinosyl glucoside, quercetin, quinic acid and rutin in the extract. Pu et al. [26] stated that quercetin and kaempferol are responsible for high FRAP values due to the number and position of hydroxyl groups at the different flavonoid skeletons. In the metal chelating assay, the PR-ME produced the highest metal chelating activity. Ferrous (Fe^2+^) ion is an unstable form of iron which contributes to formation of ROS that cause lipid peroxidation, nucleic acid or protein damage, whereas, ferric (Fe^3+^) is an inactive but more stable ion. The highest metal chelation in PR-ME is contributed to the presence of 3-*O*-caffeoylquinic acid, 5-*O*-caffeoylquinic acid, chlorogenic acid, quinic acid and quercetin. The 3-*O*-caffeoylquinic acid and 5-*O*-caffeoylquinic acid possess ideal structure chemistry for free radical scavenging activities because they have phenolic hydroxyl groups that are prone to donate a hydrogen atom or an electron to a free radical and also extended conjugated aromatic system to delocalize an unpaired electron. According to Luzia et al. [27], 5-*O*-caffeoylquinic acid could act as a metal chelator due to presence *ortho*-hydroxy groups in its chemical structure. The fewer the number OH groups, the lower the probability of hydrogen loss and the lower the probability of oxidation of the flavonoid and the reduction of the metal [27]. Quercetin is a known iron chelator and has iron stabilizing properties. The chemical structure of quercetin contains a catechol moiety in the B ring that participates in metal chelation [28,29]. The present study exhibited the highest percentage of hydrogen peroxide scavenging activity in the PR-ME extract. The scavenging activity of hydrogen peroxide radical by PR-ME is due to the presence of compounds which were dr detected by LCMS/MS including 3-*O*-caffeoylquinic acid, kaempferol and quercetin. The scavenging of hydrogen peroxide by plant extracts may be attributed to their phenolic contents, which donate electrons to hydrogen peroxide and reduce it to water [30]. Hydrogen peroxide is necessary for peroxidase- catalyzed reactions and its scavenging could be the cause of a lower enzymatic oxidative process. The nitric oxide (NO) radical is involved in several pathological diseases, such as chronic inflammation, autoimmune diseases and rheumatoid arthritis The NO scavenging activity was found to increase with increasing concentration of extracts. The present study showed that PR-ME inhibited the nitrite formation by directly competing with oxygen in the reaction with nitric oxide. The high inhibition of PR-ME be due to the presence of 3-*O*-caffeoylquinic acid, 5-*O*-caffeoylquinic acid, quinic acid, quercetin, and kaempferol. In fact, 5-*O*-caffeoylquinic acid, quercetin and kaempferol have already revealed antioxidant capacity in several systems [31,32]. Quercetin was claimed as the most potent flavonoid scavenger of ROS, including RNS like NO [33,34]. In fact, quercetin not only prevents the occurrence of oxidative stress but also helps mitigate inflammation [35,36,37]. Indeed, it has already been shown that quercetin and kaempferol are able to inhibit NO production by acting against NOS isoforms [38]. Furthermore, quercetin also inhibits ROS/RNS generation, inducible nitric oxide synthase expression and NF-κB activation in IL-1b-activated rat hepatocytes [39]. The superoxide scavenging activity of extracts from *P. rubra* red flowers was increased markedly with the increase in concentration. The present study shows that all the extracts from *P. rubra* flowers can potentially act as superoxide scavengers except the hexane extract. Moreover, the good inhibitory effects of the PR-ME of *P. rubra* on superoxide anion formation noted herein possibly render it a promising antioxidant. The highest scavenging activity against superoxide radical in PR-ME was due to the presence of phytochemical compounds which were detected by LCMS/MS. Several of these compounds have been reported as superoxide scavengers in previous research. The hydroxyl group at C-3′ and C-4′ of the B ring in rutin, kaempferol and quercetin should contribute to the superoxide radical scavenging activity due to hydrogen donation activity [40]. Nevertheless, the efficiency of superoxide scavenging could be decreased by blocking of hydroxyl groups by sugars or alkoxyl substituents through glycosylation of quercetin and or kaempferol [41]. The present study indicated that PR-ME was more efficient in scavenging of superoxide anion activity as compared to a quercetin standard due to the present of superoxide scavenging activity of 3-*O*-caffeoquinic acid, 5-*O*-caffeoquinic acid, quinic acid, quercetin, kaempferol and rutin compounds. As the XO enzyme was inhibited, it reduced the XO activity and this would then eventually inhibit the catalysis of hypoxanthine to xanthine, then xanthine to uric acid. Thus, the inhibition of XO enzyme will reduce the production of uric acid. The higher XO inhibition of PR-ME in the in vitro assay could be due to the presence of 3-*O*-caffeolquinic acid, chlorogenic acid, quercetin, kaempferol and rutin as these phytochemicals were detected by LCMS/MS. Several studies have reported that rutin, kaempferol and quercetin inhibit the activity of XO enzyme [5,42,43,44]. Masuoka et al. [45] have described that the XO inhibition activity of kaempferol 3-*O* glucosides was lower compared to its aglycone due to a competing xanthine binding site in xanthine oxidase. In addition, Cos et al. [5] stated that a flavonoid chemical that contains hydroxyl groups at C5 and C7 and a double bond between C2 and C3 is essential for a high inhibitory activity on xanthine oxidase. Nagao et al. [43] stated that hydroxyl groups at the C5 and C7 of flavonoids was essential for inhibition of xanthine oxidase activity, but not a hydroxyl group at C3 of the flavone structure of quercetin. Furthermore, kaempferol is not only capable of inhibiting XO activity but also possesses superoxide scavenging activity [5]. The present results showed that the XO inhibition activity could be linked to the content and the nature of flavonoids and phenolic compounds present in the extracts. In fact, the present result was in agreement with other researchers indicating that a possible synergy between polyphenols and other components present in methanol extracts might contribute to their overall antioxidant activity [46,47].

Generally, the present study indicated that PR-ME of *P. rubra* produced the highest XO inhibition activity and also in the superoxide radical scavenging assay. In fact, the inhibition activity of XO enzyme was higher compared to superoxide scavenging activity. Thus, the PR-ME extract could be categorized as a XO inhibitor with additional superoxide scavenging activity. Cos et al. [5] reported similar findings for flavonoid compounds. Matsuoka et al. [45] have postulated the possible mechanism pathway for compounds that are superoxide radical antioxidants and XO activity inhibitors. The PR-ME showed strongest the XO inhibition activity in in vitro model suggesting that there is strong correlation with its ethnomedicinal use in the treatment of inflammatory disorders and rheumatic diseases. In the animal study, the acute toxicity test showed that no lethality or any toxic reactions were observed at a dose of 4000 mg/kg body weight. The anti-hyperuricemic activity and XO inhibitory activity of PR-ME extract were evaluated in an in vivo model using doses of 200 mg/kg and 400 mg/kg body weight. The body weights of the eight groups of animals increased gradually through the 7 days of treatment. The rats in each group had lustrous body hair, normal water and diet consumptions, and urine volume and were in good mental state during the experimental period, indicating that PR-ME extract did not cause any side effects on the rats. The result demonstrated that PR-ME significantly affected serum urate levels in hyperuricemia-induced rats but not in normal rats after 7 days of treatment. The administered doses of PR-ME (400 mg/kg and 200 mg/kg) were shown to elicit a dose-dependent decrease in serum uric acid levels only in hyperuricaemia-induced rats but not in the non-induced rats. Several studies have reported that most medicinal plant extracts exhibit less inhibitory effects on serum uric acid levels in normal mice compared with animals pre-induced with PO [14,48,49,50]. Thus, the phytochemical compounds in the methanol extract detected by our LCMS/MS analysis did not exhibit a significant reduction in uric acid levels in normal rats within 7 days of administration. The serum urate level of hyperuricemia- induced rats were decreased significantly in a dose dependent manner with doses of 200 and 400 mg/kg of PR-ME. The uric acid-lowering effect of group 6, which was hyperuricemia-induced rats treated with a high dose of 400 mg/kg PR-ME and group 7 which was hyperuricemia-induced rats treated with a low dose of 200 mg/kg PR-ME indicated that the PR-ME has the potential to attenuate hyperuricemia. As compared to the standard allopurinol, known for its protective role as a XO inhibitor showed that it was effective in reducing 78.2% of the uric acid. The decrease of uric acid levels in serum could be due to the presence of the compounds quercetin, kaempferol and rutin in PR-ME extract as detected in LCMS/MS. In fact, kaempferol, quercetin and rutin were capable of reducing the accumulation of purine metabolites in blood following PO-induction rats as reported by several researchers [14,51,52,53].

The inhibition effects on XO activity were more dominant in the groups of hyperuricemia-induced rats compared to their effects on normal rats. The XO enzyme activity in serum was correlated with XO enzyme activity in liver in the hyperuricemic rats. The present study showed that the PR-ME extract exerted an inhibitory action on induced XO in both serum and liver of hyperuricemia-induced rats in a dose-dependent manner. The presence of 3-*O*-caffeolquinic acid, 5-*O*-caffeoylquinic acid, chlorogenic acid, kaempferol, quercetin and rutin contributed to the beneficial effects of PR-ME extract on the inhibition of XO enzyme activities. Zhu et al. [14] have described that quercetin and rutin are capable of inhibiting XO enzyme in in vitro and in vivo models.

The present results indicated that the PR-ME extract possesses good antioxidant activity via different mechanisms, including hydrogen atom donation, the ability to reduce ferric ions, metal chelating effect, hydrogen peroxide scavenging ability, nitric oxide scavenging ability and superoxide anion scavenging ability. Moreover, the PR-ME showed potential XO inhibitory activity in in vitro assay and in vivo in both serum and liver of PO-induced hyperuricemic rats. The reducing effects of serum urate levels from PO-induced hyperuricemia was mediated to a XO inhibitory activity of the PR-ME extract. There is a relationship between oxidative stress and gout disease, as XO enzyme contributes to the production of uric acid by catalyzing the transformation of hypoxanthine into xanthine, with concomitant production of superoxide and hydrogen peroxide radicals [1,9,54]. Thus, the results of this research have shown that the bioactive compounds detected in the PR-ME extract of *P. rubra* flowers are capable of inhibiting the activity of XO enzyme.

## 4. Materials and Methods

### 4.1. Plant Materials

The flowers of *P. rubra* were collected at the Faculty of Science, University of Malaya. The authenticity of the sample was verified by the plant taxonomist Professor Dr Ong Hean Choi of the Institute of Biological Science, University of Malaya. A voucher specimen with reference no. KLU 48177 was deposited in the University of Malaya Rimba Ilmu Herbarium. 

### 4.2. Preparation of the Flower Extract

The flower samples 50 gm were soaked overnight with hexane, chloroform and methanol (200 mL). The extracts were filtered and concentrated using a vacuum rotary evaporator. 

### 4.3. Detection of Phytochemical Compounds with Liquid Chromatography Mass Spectrometry (LCMS)

The methanol flower extract of *P. rubra* was analyzed with Liquid Chromatography Mass Spectrophotometry (LCMS) to determine the chemical compounds present. Analyses were run on an AB Sciex 3200QTrap LCMS/MS instrument (Agilent Technologies, Santa Clara, CA, USA) as full scans with MS/MS data collection in negative ionization mode. A Aqua C18 (50 mm × 2.0 mm × 5 μM, Phenomenex, Torrance, CA, USA) column was used in a rapid screening using a 15 min run time.

### 4.4. Determination of Total Phenolic Content

The total phenolics content was determined using Folin-Ciocalteu (FC) reagent according to the method described by [55] with slight modifications. Briefly, 20 μL of red flower from *P. rubra* extract was mixed with 100 μL of FC reagent (diluted 10-fold with distilled water) in a 96-well microplate, incubated for 5 min, and 75 μL of Na_2_CO_3_ solution (7.5%) was added. After 2 h of incubation period in darkness at room temperature, the absorbance was measured at 740 nm using a microplate reader (Sunrise, Austria). Gallic acid (1 mg/1 mL) was used as standard for calibration and construction of a linear regression line and DMSO 3% was used as a blank. The total phenolic content of the extract was calculated as mg gallic acid equivalent (GAE) mg/g of dry weight extract and were done in triplicate.

### 4.5. Determination of Total Flavonoid Content

The total flavonoid content was determined according to the method described by [56] with slight modifications. Briefly, 50 μL of extracts were added with 70 μL of distilled water and 15 μL of 5% NaNO_2_ solution in a 96-well microplate. The solutions were well mixed and incubated for 5 min at room temperature. Then, 15 μL of 10% AlCl_3_ solution was added into the mixture. After 6 min of incubation, 100 μL of 1M NaOH solution was added, and the absorbance was measured at 510 nm with a microplate reader (Sunrise, Austria). Methanol was used as blank. The final absorbance of each extracts was compared with a plotted quercetin standard curve. The total flavonoid content of the extracts was expressed in mg quercetin equivalent (QE) mg/g of dry weight extract.

### 4.6. Determination of Antioxidant Activity

#### 4.6.1. 2,2-Diphenyl-1-picrylhydrazyl (DPPH) Scavenging Activity Assay

The quantitative measurements of radical scavenging assay were carried out according to the method as described by [57] with slight modifications. Briefly, 40 μL of extracts of each concentration (15.63 to 250 μg/mL) were mixed with 200 μL of 50 μM DPPH solution in methanol. The mixture was immediately shaken and incubated for 15 min in the dark at room temperature. The absorbance reading was measured at 517 nm using an ELISA microplate reader (Tecan Sunrise). Ascorbic acid (15.63–250 μg/mL) served as a standard positive control and the negative control was methanol.

#### 4.6.2. Ferric Reducing Anti-Oxidant Power (FRAP)

The FRAP assay was carried out in 96-well microplates as described according to the method of Benzie and Strain [58] with slight modifications. Twenty microliters of red flowers of *P. rubra* extracts in ethanol were mixed with 200 μL of daily prepared FRAP reagent, which contained 5 mL 10 mM TPTZ in 40 mM HCl, 5 mL of 20 mM FeCl_3_, and 50 mL of 0.3 M acetate buffer (pH 3.6) in 96-well microplate. After 8 min of incubation time, the formation of the TPTZ-Fe^2+^ complex in the presence of antioxidant compounds in the extract was measured at 595 nm with a microplate reader (Tecan Sunrise). Methanol was used as a blank. Ferrous sulfate (FeSO_4_) solution (0.2 mM to 1 mM) was used for standard calibration curve. The FRAP value was evaluated according to the linear regression between standard solutions and absorbance at 595 nm and the results were estimated as mmol Fe^2+^/g of dry extract from triplicate tests.

#### 4.6.3. Metal Chelating Activity Assay

The ferrous ion chelating activity of the flowers extract of *P. rubra* extracts was determined according to the procedure as described by Srivastava et al. [59] with slight modifications. Briefly, 100 μL of extracts of each concentration (15.63–250 μg/mL) were mixed with 120 μL distilled water and 10 μL FeCl_2_ (2 mM) in a 96-well microplate. Ferrozine (FZ, 5 mM, 20 μL) was added to the mixture to initiate the reaction. The reaction mixture was incubated at room temperature for 20 min and absorbance at 562 nm. EDTA was served as positive control (15.63–250 μg/mL) while 0.05% DMSO was used as a negative control; blank was without FZ (20 μL of distilled water instead of FZ). The percent inhibition of Fe^2+^-FZ complex formation was calculated. The concentration of extracts required to chelate 50% of the Fe^2+^ ion (IC_50_) was calculated from the graph against the percentage of inhibition.

#### 4.6.4. Hydrogen Peroxide Scavenging Activity Assay

The scavenging capacity for hydrogen peroxide was measured according to the method as described by Sroka and Cisowki [60] with slight modifications. A solution of H_2_O_2_ (2 mM) was prepared in 50 mM phosphate buffer (pH 7.4). H_2_O_2_ concentration was determined spectrophotometrically at 230 nm absorption using the molar extinction coefficient for H_2_O_2_ of 81 mol^−1^cm^−1^. 0.1 mL of each concentrations of extracts and ascorbic acid (16.63–250 μg/mL in respective solvents), was transferred into the test tubes and their volumes were made up to 0.4 mL with 50 mM phosphate buffer (pH 7.4) or solvent (methanol). After addition of 0.6 mL H_2_O_2_ solution, tubes were vortexed and absorbance was measured at 230 nm was determined after 10 min, against a blank. 50 mM phosphate buffer without H_2_O_2_ was used as blank.

#### 4.6.5. Nitric Oxide Scavenging Activity Assay

The nitric oxide scavenging activity of the flowers extract of *P. rubra* was determined according to the procedure of Srivastava et al. [23] with slight modifications. Briefly, 50 μL of extract at each concentration (15.63–250 μg/mL) and an equal amount of sodium nitroferricyanide (Na_2_[Fe(CN)_5_NO]·2H_2_O, 10 mM) in phosphate-buffered saline (20 mM, pH 7.4) were mixed well in a 96-well microplate. The mixture was incubated at room temperature for 150 min and 125 μL of Griess reagent was added. After 10 min, the absorbance was measured at 546 nm with a microplate reader (Tecan Sunrise). Curcumin (15.63–250 μg/mL) and ethanol were used as a standard and control, respectively. The reaction mixture without Griess reagent served as blank. 

#### 4.6.6. Superoxide Scavenging Activity Assay

The superoxide scavenging activity was determined by PMS-NADH with slightly modifications as described by Shukla et al. [61] with slight modifications. Briefly, 50 μL of NBT solution (0.2 mM in distilled water) were mixed with 50 μL of NADH solution (0.5 mmol/L in 0.1 M Tris-HCl, pH 8.0) and 100 μL of extract at each concentrations (15.63–250 μg/mL) and treated with 50 μL of PMS solution (25 μM PMS in distilled water). The reaction mixture was incubated at room temperature for 10 min, and the absorbance at 570 nm was measured. Quercetin and ascorbic acid were used as positive control.

### 4.7. Xanthine Oxidase Inhibitory Activity via In Vitro System

The in vitro XO inhibitory assay of the flower extract of *P. rubra* was performed according to the procedure as described by Owen and Johns [62] with slight modifications. Briefly, 1 mL of extract at each concentration (62.5 to 500 μg/mL) was added to 2.9 mL of phosphate buffer, and 0.1 mL of enzyme solution (0.01 units/mL in phosphate buffer, pH 7.5) which was prepared immediately before use. After pre-incubation at 25 °C for 15 min, the reaction was initiated by the addition of 2 mL of 150 mM xanthine solution in the same buffer which acts as a substrate and this reaction mixture were incubated at 25 °C for 30 min. The reaction was stopped by addition of 1 mL of 1 N of HCl and the absorbance was measured at 290 nm by using a UV spectrophotometer. Blank was prepared in the same way, but the enzyme solution added to the assay mixture after adding 1 N HCl. The assay was done in triplicate. One unit of XO is defined as the amount of enzyme required to produce 1 mmol of uric acid per min at 25 °C.

### 4.8. Animal Study

Male Sprague Dawley (SD) rats (200 ± 30 g) were purchased from the University of Malaya Animal Experimental Unit. Rats were allowed to adapt to their environment before being used for experiments for at least 1 week. They were maintained in a room controlled at 22–24 °C with a relative humidity of 60 ± 5% and a 12 h light/dark cycle (6:00 a.m.–6:00 p.m.). They were given standard chow and water ad libitum for the duration of the experiment. All experimental protocols described in this study were approved by Ethics Committee on Animal Experiment of University of Malaya Animal Care and Use Committee with Ethics Approval No. ISB/12/09/2014/SSPMI (R).

### 4.9. Acute Toxicity Study

An acute toxicity study of PR-ME extract of red flower *P. rubra* was performed in healthy adult Sprague Dawley (SD) rats according to the OECD 423 guidelines. Observations were made on any changes in skin, fur, eyes and mucous membranes, and also respiratory, autonomic including defecation and urination, the central nervous systems including spontaneous reaction, reactivity, touch response, pain response, and the behavior pattern including alertness, restlessness and irritability. The biological evaluation is carried out at 1/10th of the maximum tolerated dose.

### 4.10. In Vivo Study of Hyperuricemic Rats

The hyperuricemia effect of the flower methanol extract of *P. rubra* was carried out following the methods described by Liu et al. [63] with slight modifications. Hyperuricemia was induced in the rats by intraperitoneal (i.p.) administration of potassium oxonate (PO) at 280 mg/kg body weight. Briefly, the eight groups of SD rats are assigned to this experiment (*n* = 6 per group). Generally, groups 1, 2 and 3 are the normal control groups which are not induced by PO. Group 1 served as a baseline which do not received any solvent vehicle and treatment within seven days of experimentation. Group 2 served as a normal rat vehicle control group which was only orally administered with 0.5% CMC solution for seven consecutive days. In group 3, the rats were only administered the extract of *P. rubra* at high dose of 400 mg/kg body weight whereas in group 4 with low dose of 100 mg/kg body weight was used. Group 5 is the hyperuricemia group that served as gout control which received intraperitoneal administration of PO at a dose of 280 mg/kg without receiving any treatment. The groups 6 and 7 were given flower methanol extract only at high dose of 400 mg/kg body weight and low dose (200 mg/kg body weight) respectively. The group 8 was given the standard drug allopurinol at 10 mg/kg body weight. The treatments for groups 6, 7 and 8 were given 1 h after induction with PO. On the seventh day, two hours after PO-induction, the rats were anaesthetized with ketamine and xylazine at 100 and 20 mg/kg, respectively, via intraperitoneal injection and whole blood samples was collected by cardiac puncture 1 h later after final drug administration. The blood was allowed to clot for 1 h at ambient temperature and centrifuged at 3500 rpm for 5 min to obtain the serum.

### 4.11. Body Weight Measurement of Rats

The fasting body weight (BW) of rats were monitored every 2 days until final administration of the flower sample.

### 4.12. Determination of Uric Acid Assay

The serum uric acid concentration was determined by an enzymatic colorimetric method, using a standard diagnostic kit (MAK077) purchased from Sigma-Aldrich (St. Louis, MO, USA) according to manufacturer’s instructions.

### 4.13. Determination of Xanthine Oxidase Assay in In Vivo Model

The rats livers were excised immediately after blood collection, washed in 0.9% cold saline and rapidly stored at −80 °C until further experiment. Briefly, livers were homogenized in 4 mL of 80 mM sodium phosphate buffer (pH 7.4) and, then, the homogenate was centrifuged at 3500× *g* for 10 min at 4 °C. Lipid layer was carefully removed, and supernatant was further centrifuged at 10,000× *g* for 60 min at 4 °C. The final supernatant was used for xanthine oxidase enzyme activity assays. Xanthine oxidase activity was determined spectrophotometrically using a standard kit (MAK078) purchased from Sigma-Aldrich.

## 5. Statistical Analysis

The results were expressed as the mean ± standard error (SEM) for the three independent experiments. Differences between extracts were analyzed by one way ANOVA followed by Duncan and Dunnett’s post hoc multiple comparison test at the 5% level (*p* < 0.05). The statistical program SPSS version 22.0 (SPSS Inc., Chicago, IL, USA) was used in the entire test.

## 6. Conclusions

The analysis of the PR-ME extract *P. rubra* by liquid chromatography-tandem mass spectrometry (LCMS/MS) indicated the presence of 3-*O*-caffeoylquinic acid, 5-caffeoylquinic acid, 1,3-dicaffeoquinic acid, chlorogenic acid, citric acid, 3,3-di-*O*-methylellagic acid, kaempferol-3-*O*-glucoside, kaempferol-3-rutinoside, kaempferol, quercetin 3-*O*-α-l-arabinopyranoside, quercetin, quinic acid and rutin. The PR-ME extract showed high total phenol, flavonoid content and strong DPPH scavenging activity, ferric reducing power, chelating activity, hydrogen peroxide radical scavenging activity, nitric oxide radical scavenging activity and superoxide radicals scavenging activity. It showed no toxic effect at a dose of 4000 mg/kg and no lethality or any toxic effects were observed in the experimental animals during the entire experimental period. A reduction of serum uric acid level in a dose dependent manner in hyperuricemia-induced rats but not in the normal rats was observed. There was no significant difference in body weight between treated rats and normal rats. The antihyperuricemic activity of the PR-ME extract was mediated by XO inhibition. The inhibition activity of XO enzyme in vitro and in vivo, antihyperuricemic effect in serum and antioxidant activity of *P. rubra* was due to presence of the phytochemicals detected and identified by LCMS/MS. Thus, the results of this study indicated that red flower of *P. rubra* extract has highly potential to be used for the treatment of gout and hyperuricemia-related diseases.

## Figures and Tables

**Table 1:** TPC and TFC Values of *P. rubra* flower extracts.

Extracts of *P. rubra*	Total Phenolic Content (mg GAE)/g Dry Extract	Total Flavonoid Content (mg QE)/g Dry Extract
PR-HE	2.21 ± 0.76 ^a^	1.20 ± 1.16 ^a^
PR-CE	66.21 ± 1.27 ^b^	119.20 ± 2.40 ^b^
PR-ME	184.63 ± 0.77 ^c^	203.20 ± 1.76 ^c^
PR-WE	110.79 ± 1.07 ^d^	163.20 ± 1.33 ^d^

Each values is presented as mean ± SE (*n* = 3). The means with different lowercase letters (a, b, c and d) in the same column are significantly different at *p* < 0.05 (ANOVA, followed by Duncan’s multiple comparison test). Gallic acid equivalent (GAE); Quercetin equivalent (QE).

**Table 2:** IC_50_ and FRAP values of antioxidant activities of *P. rubra* flower extracts.

Extracts/Positive Control	DPPH (IC_50_ μg/mL)	FRAP (mmol Fe^2+^/g Extract)	Metal Chelating (IC_50_ μg/mL)	Hydrogen Peroxide (IC_50_ μg/mL)	Nitric Oxide (IC_50_ μg/mL)	Superoxide (IC_50_ μg/mL)
PR-HE	ND	0.220 ± 0.003 ^a^	ND	ND	ND	ND
PR-CE	ND	0.973 ± 0.004 ^b^	ND	ND	ND	249.61 ± 0.01 ^c^
PR-ME	59.39 ± 0.03 ^a^	4.95 ± 0.04 ^c^	185.56 ± 0.01 ^a^	248.38 ± 0.04 ^a,b,c^	62.10 ± 0.01 ^a^	54.77 ± 0.04 ^a,d^
PR-WE	231.06 ± 0.02 ^b,c^	3.50 ± 0.03 ^d^	248.96 ± 0.03 ^b^	ND	116.67 ± 0.02 ^b^	98.56 ± 0.04 ^b,e^
Ascorbic acid	29.60 ± 0.02 ^d^	_	_	104.07 ± 0.01 ^a,b,c^	_	50.52 ± 0.01 ^a,d^
EDTA	_	_	56.157 ± 0.004 ^c^	_	_	_
Curcumin	_	_	_	_	24.81 ± 0.01 ^c^	_
Quercetin	_	_	_	_	_	97.14 ± 0.01 ^b,e^

Each value in the table is presented as mean ± SE (*n* = 3). The means with different lowercase letters (a, b, c and d) in the same column are significantly different at *p* < 0.05 (ANOVA, followed by Duncan’s multiple comparison test). ND: not detected.
